# Unbiased characterization of genotype-dependent metabolic regulations by metabolomic approach in *Arabidopsis thaliana*

**DOI:** 10.1186/1752-0509-1-53

**Published:** 2007-11-21

**Authors:** Miyako Kusano, Atsushi Fukushima, Masanori Arita, Pär Jonsson, Thomas Moritz, Makoto Kobayashi, Naomi Hayashi, Takayuki Tohge, Kazuki Saito

**Affiliations:** 1 RIKEN Plant Science Center, 1-7-22, Yokohama, Kanagawa 230-0045, Japan; 2 Department of Computational Biology, Graduate School of Frontier Sciences, University of Tokyo, Kashiwa, Chiba 277-8561, Japan; 3 Group for Chemometrics, Organic Chemistry, Department of Chemistry, Umeå University, SE-901 87 Umeå, Sweden; 4 Umeå Plant Science Centre, Department of Forest Genetics and Plant Physiology, Swedish University of Agricultural Sciences, SE-901 87 Umeå, Sweden; 5 Department of Molecular Biology and Biotechnology, Graduate School of Pharmaceutical Sciences, Chiba University, Chiba 263-8522, Japan; 6 Max-Planck Institut für Molekulare Pflanzenphysiologie, Am Mühlenberg 1, 14476 Golm-Potsdam, Germany

## Abstract

**Background:**

Metabolites are not only the catalytic products of enzymatic reactions but also the active regulators or the ultimate phenotype of metabolic homeostasis in highly complex cellular processes. The modes of regulation at the metabolome level can be revealed by metabolic networks. We investigated the metabolic network between wild-type and 2 mutant (*methionine-over accumulation 1 *[*mto1*] and *transparent testa4 *[*tt4*]) plants regarding the alteration of metabolite accumulation in *Arabidopsis thaliana*.

**Results:**

In the GC-TOF/MS analysis, we acquired quantitative information regarding over 170 metabolites, which has been analyzed by a novel score (ZMC, z-score of metabolite correlation) describing a characteristic metabolite in terms of correlation. Although the 2 mutants revealed no apparent morphological abnormalities, the overall correlation values in *mto1 *were much lower than those of the wild-type and *tt4 *plants, indicating the loss of overall network stability due to the uncontrolled accumulation of methionine. In the *tt4 *mutant, a new correlation between malate and sinapate was observed although the levels of malate, sinapate, and sinapoylmalate remain unchanged, suggesting an adaptive reconfiguration of the network. Gene-expression correlations presumably responsible for these metabolic networks were determined using the metabolite correlations as clues.

**Conclusion:**

Two Arabidopsis mutants, *mto1 *and *tt4*, exhibited the following changes in entire metabolome networks: the overall loss of metabolic stability (*mto1*) or the generation of a metabolic network of a backup pathway for the lost physiological functions (*tt4*). The expansion of metabolite correlation to gene-expression correlation provides detailed insights into the systemic understanding of the plant cellular process regarding metabolome and transcriptome.

## Background

Metabolomics, the chemical profiling of (all) cellular metabolites by their identification and quantification, is a rapidly expanding strategy in the post-genomics era complementing transcriptomics and proteomics thereby constituting a trilogy. Metabolomics is regarded as the most promising among the 3 strategies particularly in plant science because plants have large and often polyploid genomes that seriously impede traditional genomic, transcriptomic, and proteomic approaches [1,2]. Recent technological advances in mass spectrometry have realized reliable and highly sensitive measurements of metabolites. Indeed, metabolomics has been utilized not only to investigate plant metabolism *per se *but also to identify unknown gene functions by comparing the profiles between wild-type and genetically altered plants or during developmental changes [3-7]. The popular metabolomics strategy is to focus on the pattern of metabolite concentrations under the given conditions. Such quantitative information on metabolites has been used either to predict gene functions directly involved in metabolic processes [8-11], to delineate metabolism and its regulatory networks [12-14], or to distinguish metabolic phenotypes [15-17].

Metabolome changes are highly sensitive to the fluctuations in biological conditions as compared to transcriptome changes [3,12,18]. This is an intrinsic nature of metabolome as an ultimate cellular phenotype, and such small fluctuations in the metabolome across independent plants may provide information regarding the formation of metabolic networks [12,18].

Although there is a large potential to use vast metabolome datasets for the elucidation of metabolic networks, only limited datasets are publicly available. This is strikingly different from transcriptomics, in which a multitude of data for *Arabidopsis thaliana*, the most widely studied model plant, is available in the public domain, e.g., AtGenExpress [19], AthCoR@CSB.DB [20], Genevestigator [21], and ATTED-II [22]. Thus, previous metabolomics studies simply identified specific metabolite changes in order to explain a particular biological process under the given conditions. The current challenges are to figure out more detailed networks involving all the molecular elements (metabolite, protein, and transcript) in a global manner across a variety of biological conditions.

In the present study aiming to computationally elucidate metabolic regulation in an unbiased manner, we focus on not only the changes in metabolite concentrations but also the metabolite correlations in *A. thaliana *mutants by means of gas chromatography time-of-flight mass spectrometry (GC-TOF/MS). We selected 2 representative mutants, namely, *methionine-over accumulation 1 *(*mto1*) and *transparent testa4 *(*tt4*) for primary and secondary metabolism, respectively. The *mto1 *mutant, caused by the lesion of the feedback regulation in methionine biosynthesis, is known to accumulate from 10- to 40-fold more soluble methionine than the wild-type (WT) plant with few differences in other amino acid levels [23,24]. The *tt4 *mutant is deficient in a chalcone synthase (CHS) gene and thus cannot produce flavonoids – typical plant secondary products that function as protectants against abiotic stresses such as UV light [25]. This mutant is presumed to undergo various physiological changes triggered by flavonoids. Since both the mutants reveal a so-called "silent phenotype" exhibiting no visible changes in development under normal growth conditions, these mutants would be ideal for the investigation of metabolotypes caused solely by a single genetic alteration and protected from secondary effects due to a developmental abnormality.

In general, interpretation of metabolite concentration is not straightforward. Higher concentration of a metabolite, for example, may result from very high metabolic flux, accelerated production, decelerated degradation, or their combinations. One method to distinguish such flux modes is to look at metabolite correlations; from high correlation in metabolite concentrations we can hypothesize, although not conclusively, their co-existence in common metabolic pathways or coordinated regulation due to some biological mechanisms. Indeed, metabolite correlations have been used in finding bottlenecks or metabolic shifts on pathways [14], characterizing physiological response to environmental changes [13], or simply as fingerprints of the underlying physiological states [12,18]. In the current analysis, we integrate metabolite correlation data with publicly available gene-coexpression data to obtain insights on the relationship between metabolites and gene regulations. This approach can relate metabolite correlation, at least partly, with the hidden coexpression of genes and lead us to the discovery of novel metabolite-gene networks.

## Results

### Metabolic profiles vary between WT, *mto1 *and *tt4 *mutants

The metabolome of the aerial regions of individual WT plants and the *mto1 *and *tt4 *mutants were analyzed by GC-TOF/MS. The obtained raw data were imported to the MATLAB software for peak alignment and for extraction of spectra using a hierarchical multivariate curve resolution (H-MCR) method as described previously [26,27]. By this method, we could obtain faster a correct data table with peak areas, including corresponding mass spectra, than by commercial software often used for metabolome analysis [28]. The H-MCR process extracted 518 metabolite peaks and their mass spectra. Using the national institute of standards and technology (NIST02) and our custom software for spectral annotation (Fukushima *et al*., in preparation), 93 peaks were identified or annotated as known metabolites. In addition, 78 peaks were annotated with mass spectral tags (MSTs) [29,30]. These peaks were consistently observed though not identified completely.

**Figure 1:**
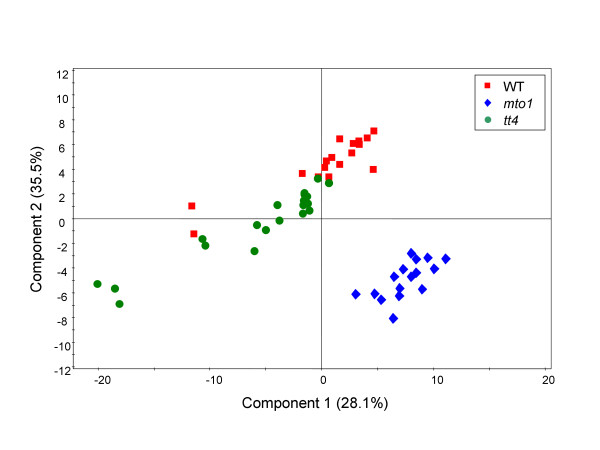
**The partial least square-discriminate analysis (PLS-DA) score scatter plot of the first 2 components for WT and 2 mutants, *mto1 *and *tt4***. Dotted circles represent individual plant samples. This PLS-DA analysis represents the differentiation of 53 samples (WT, *n *= 17; *mto1*, *n *= 16 [13 samples from biological replicates and 3 samples from analytical replicates]; and *tt4*, *n *= 20). The symbols correspond as follows: red square, wild-type (WT); blue diamond, *methionine-over accumulation 1 *(*mto1*); green circle, *transparent testa4 *(*tt4*). The PLS-DA model shows 3 significant components according to cross-validation. The explained variation in the X-matrix (R^2^X) and the Y-matrix (R^2^Y) is 0.60 and 0.86, respectively, and the predictive ability according to 7-fold cross-validation (Q^2^Y) is 0.80.

For a total of 171 annotated peaks, partial least square-discriminate analysis (PLS-DA) was applied as a multivariate statistical analysis. The scatter plot of PLS-DA scores showed clear separation among WT, *mto1*, and *tt4 *with a good cross-validation result in accordance with their genetic background (Figure 1). The 2 mutants showed distinct metabolic snapshots that were consistent within each biological replicate of mutant plants.

Subsequently, we searched the metabolites that contributed to this separation of the mutant profiles group from those of WT (discriminative metabolites, hereafter) (Additional File 1). In order to filter the discriminative metabolites further, Welch's *t*-test was conducted for annotated peaks by interpreting the first weight vector in the PLS-DA, assuming that metabolite concentrations follow the normal distribution in the same genetic background. Between *mto1 *and WT, 34 peaks differed significantly, whereas 31 peaks were significantly different between *tt4 *and WT (*p *< 0.05). The discriminative metabolites for *mto1 *were mostly amino and organic acids, whereas sugars and the precursors of secondary products were found as discriminative metabolites for *tt4*. In *mto1*, remarkable changes in methionine-related compounds such as methionine, homocysteine, and methionine sulfone were observed (see below).

### Pronounced metabolite changes are observed in *mto1 *but fewer changes in *tt4*

**Figure 2:**
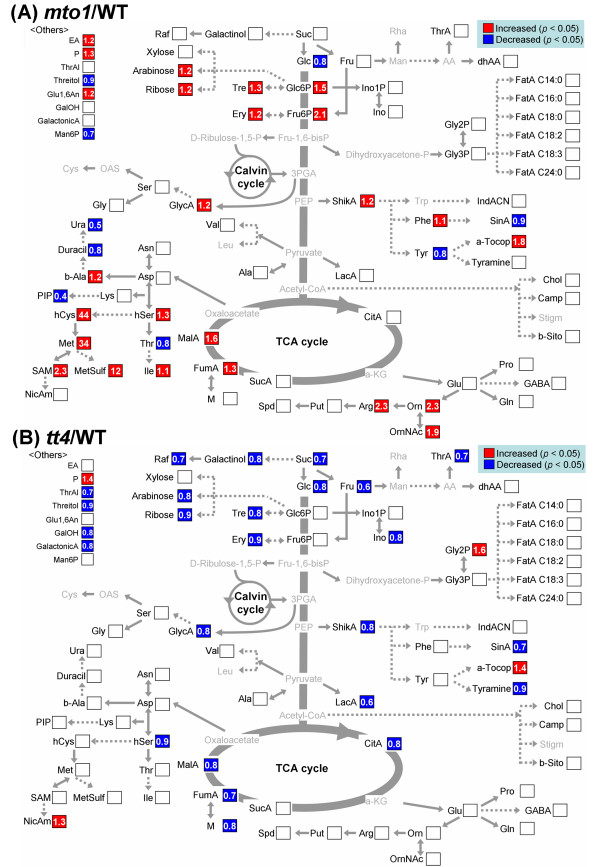
**Changes in the levels of metabolites in (A) *mto1 *and (B) *tt4 *plants**. The changes in metabolite contents were calculated by dividing the metabolite level in the mutant with that in the WT. The level of significance was set at *p *< 0.05. The metabolites with gray characters were undetectable. Abbreviations: 3-PGA, 3-phosphoglycerate; AA, ascorbate; a-KG, alpha-ketoglutarate; Ala, alanine; Arg, arginine; Asn, asparagine; Asp, aspartate; a-Tocop, alpha-tocopherol; b-Ala, beta-alanine; b-Sito, beta-sitosterol; Camp, campesterol; Chol, cholesterol; CitA, citrate; Cys, cysteine; dhAA, dehydroascorbate; Dihydroxyacetone-P, dihydroxy-acetone-phosphate; D-Ribulose-1,5-P, D-ribulose-1,5-diphosphate; Duracil, dihydrouracil; EA, ethanolamine; Ery, erythritol; FatA C14:0, *n*-tetradecanoate; FatA C16:0, *n*-hexadecanoate; FatA C18:0, stearate; FatA C18:2, linoleate; FatA C18:3, linolenate; FatA C24:0, *n*-tetracosanoate; Fru, fructose; Fru-1,6-bisP, fructose-1,6-biphosphate; Fru6P, fructose 6-phosphate; FumA, fumarate; GABA, gamma-amino-*n*-butyrate; GalactonicA, galactonate; GalacturonicA, galacturonate; GalOH, galactitol; Glc, glucose; Glc6P, glucose-6-phosphate; Gln, glutamine; Glu, glutamate; Glu1,6An, 1,6-anhydro-glucose; Gly, glycine; Gly2P, glycerol-2-phosphate; Gly3P, glycerol-3-phosphate; GlycA, glycerate; hCys, homocysteine; hSer, homoserine; Ile, isoleucine; IndACN,1*H*-indole-3-acetonitrile; Ino, *myo*-inositol; Ino1P, *myo*-inositol-1-phosphate; LacA, lactate; Leu, leucine; Lys, lysine; M, maleate; MalA, malate; Man, mannose; Man6P, mannose-6-phosphate; Met, methionine; MetSulf, methionine sulfone; NicA, nicotinic Acid; NicAm, nicotianamine; OAS, *O*-acetyl serine; Orn, Ornithine; OrnNAc, *N*-acetyl-ornithine; P, phosphate; Phe, phenylalanine; PIP, pipecolate; Pro, proline; Put, putrescine; Raf, raffinose; Rha, rhamnose; SAM, S-adenosyl-methionine; Ser, serine; ShikA, shikimate; SinaA, sinapate; Spd, spermidine; Stigm, stigmasterol; Suc, sucrose; SucA, succinate; Thr, threonine; ThrA, threonate; ThrAL, *trans*-threonic acid-1,4-lactone; Tre, trehalose; Trp, tryptophan; Tyr, tyrosine; Ura, uracil; Val, valine.

In order to identify the metabolic pathways that were modulated in terms of metabolite levels, significant metabolite changes (*p *< 0.05) observed in *mto1 *or *tt4 *against WT were compared on the metabolic map (Figure 2). Only the peaks annotated as known compounds are projected in Figure 2, while a comprehensive list of all measured peaks and the changes of these levels are shown in Additional File 2.

In *mto1*, a significant increase was observed in the levels of methionine (34-fold), homocysteine (44-fold), *S*-adenosyl-methionine (2.3-fold), and methionine sulfone (12-fold) (Figure 2A). Upregulation was also observed in the levels of ornithine, *N*-acetyl-ornithine, and arginine in the glutamate family. The levels of other amino acids in the aspartate family (aspartate, homoserine, and threonine) were nearly consistent with the reported data [23,24,31]. In contrast, there was a significant decrease in the levels of pipecolate (0.4-fold) and uracil (0.5-fold).

Less pronounced changes were observed in *tt4 *as compared to *mto1*. The levels of major sugar pools, such as sucrose, glucose, and fructose, showed a significant decrease (Figure 2B). In addition, a few components of the tricarboxylic acid (TCA) cycle and the shikimate pathway were slightly decreased in *tt4*. The levels of shikimate, sinapate, and tyramine were decreased but that of tyrosine showed no change. The levels of all the amino acids remained unchanged in *tt4*, suggesting that a lack of flavonoid biosynthesis does not largely affect the metabolism of amino acids.

### Metabolite correlations are similar between WT and *tt4 *but not *mto1*

**Figure 3:**
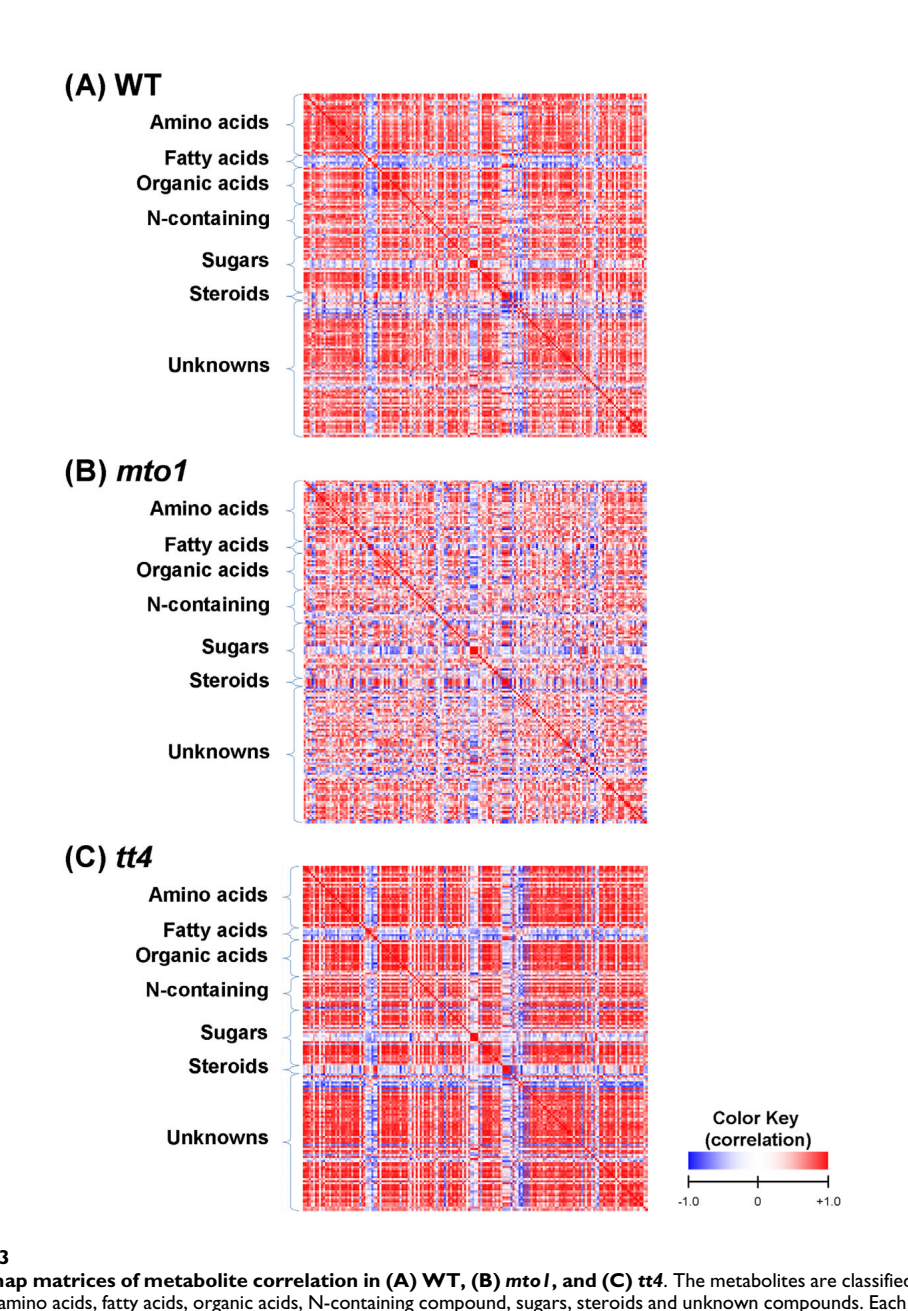
**Heat-map matrices of metabolite correlation in (A) WT, (B) *mto1*, and (C) *tt4***. The metabolites are classified into 6 groups: amino acids, fatty acids, organic acids, N-containing compound, sugars, steroids and unknown compounds. Each square indicates *r*_*Met *_(Pearson's correlation coefficient of a pair of metabolites), and the value of *r*_*Met*_is represented by the intensity of blue or red colors as indicated on the color scale.

In order to obtain insights into the regulation of metabolic network, we focused on the changes in metabolite-to-metabolite correlations in WT and the 2 mutants. Considering the small fluctuations in metabolite levels across individual plants under a given growth condition, metabolite correlations are observed as a representative metabolic profile of a given genotype [12,14,32,33]. The correlation matrices of the WT plant and the 2 mutants were visualized by heat maps (Figure 3). The numbers of significantly correlated metabolite pairs in each genotype plant (1250 in WT, 305 in *mto1*, and 3751 in *tt4*; *p *< 0.001) in Figure 3 indicated that *mto1 *lost metabolite correlations and *tt4 *strengthened correlations when compared with the WT plant, though WT and 2 mutants showed similar growth and phenotypes in the experimental condition, which was strictly controlled as much as possible. In WT and *tt4*, fatty acids had a trend of negative correlation with all the other components. In contrast, fatty acids and steroids showed positive correlations with each other. For the sugar groups in WT and *tt4*, fructose and glucose had a tendency of negative correlations with the other components; however, fructose-glucose correlated in a highly positive manner. Although *mto1 *had lesser metabolite correlations as compared to the other 2 genotypes, characteristic correlations were found only in *mto1*, e.g., linolenate (or linoleate)-steroids, linolenate (or linoleate)-nicotianamine, and glutamate-glutamine. The correlations specific to *tt4 *were also observed, such as malate-aromatic amino acids (see below).

**Figure 4:**
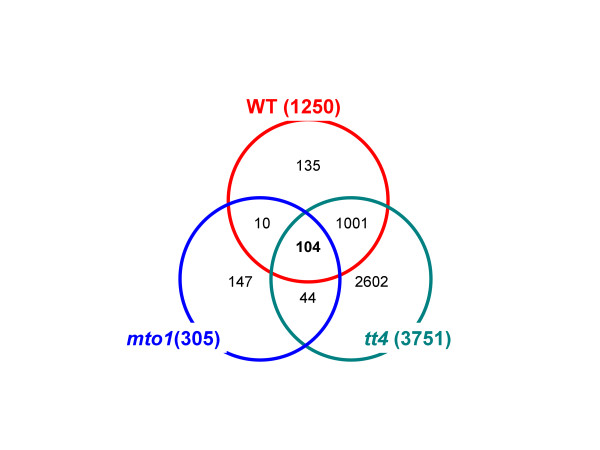
**The numbers of common metabolite correlations conserved in 3 genotypes**. The Venn diagram of the significant correlation pairs in WT, *mto1*, and *tt4*. The significance of correlations observed were determined as |*r*_*Met*_| ≥ 0.80 (*p *< 1.2 × 10^-4^, *n *= 17) for WT, |*r*_*Met*_| ≥ 0.85 (*p *< 2.3 × 10^-4^, *n *= 13) for *mto1*, and |*r*_*Met*_| ≥ 0.75 (*p *< 1.4 × 10^-4^, *n *= 20) for *tt4*.

The correlations obtained in each genotype were filtered by the statistical significance of the correlation coefficient, and these significant pairs were compared among the 3 genotypes (Figure 4). The correlation profile of *mto1 *differed largely from those of the remaining 2 genotypes. Approximately 8.3% of the significant correlation pairs were common among WT, *mto1*, and *tt4*; 88.4% (80.1% plus 8.3%) of the pairs in WT were conserved in *tt4*; only 9.1% (0.8% plus 8.3%) of the pairs were conserved between WT and *mto1*. These results suggested that the deregulated overproduction of methionine in *mto1 *strongly affected the stability of the cellular metabolite network, whereas the lack of flavonoids in *tt4 *resulted in the strengthening of metabolite correlation networks.

### Malate and aromatic compounds in shikimate pathways are tightly coregulated in *tt4*

**Figure 5:**
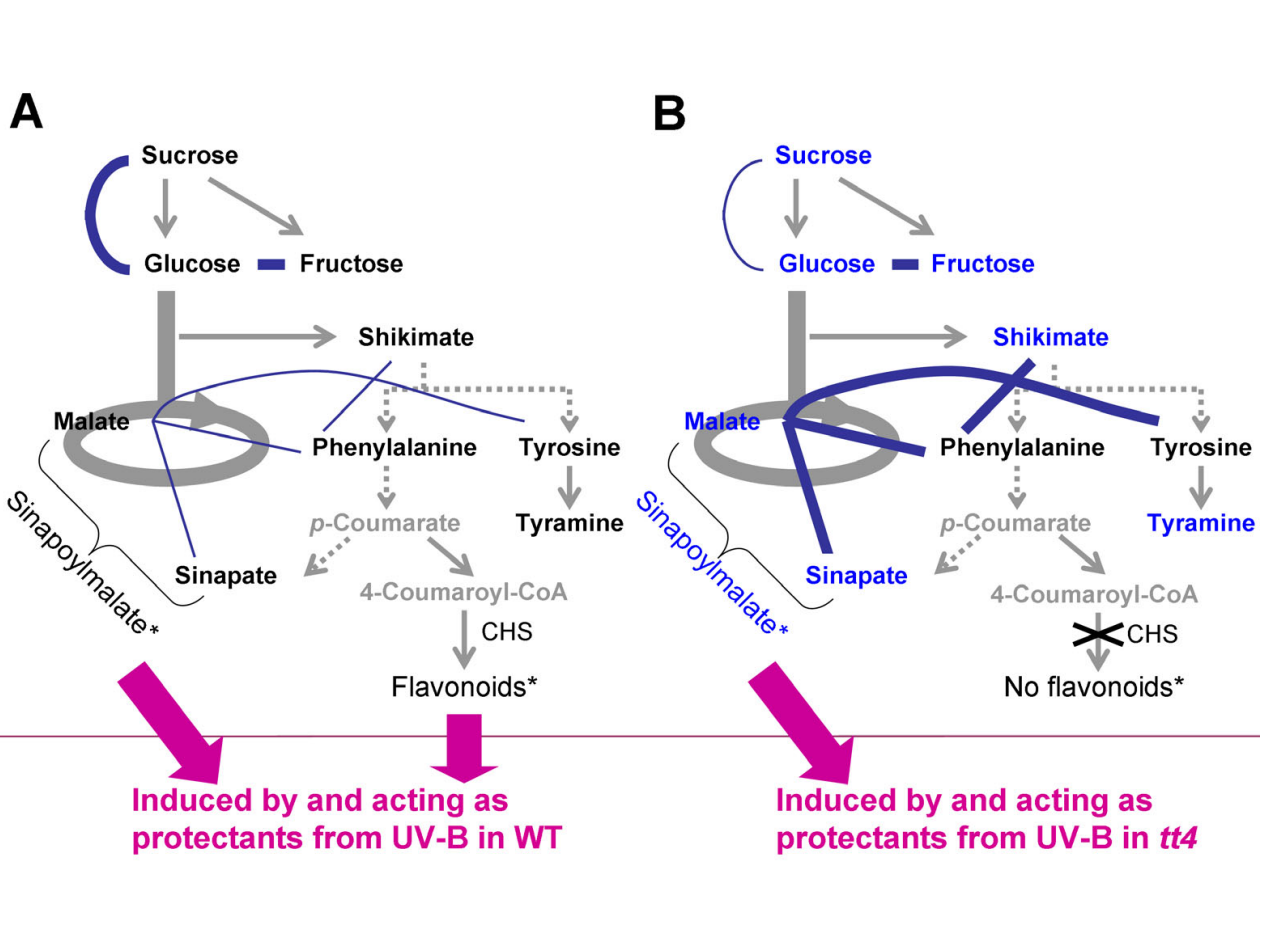
**The interconnections in the pathways of central metabolism and aromatic amino acids for (A) WT and (B) *tt4 *plants**. The metabolites whose levels changed less than 10% (*p *< 0.05) in *tt4*/WT are indicated with blue characters in (B); the metabolites with black characters in (B) had no significant changes; and the metabolites with gray characters were undetectable. The gray arrows indicate the metabolic pathways. The curved lines show correlations between metabolite pairs. The thickness of the edges between the metabolites represents the significance of correlations (*r*_*Met *_> 0.88). Although the sinapoylmalate level in *tt4 *did not increase, the correlations of malate with aromatic compounds were intensified in the *tt4 *mutant, indicating possible adaptive response to UV stress under the flavonoid-deficient *tt4 *mutant by reconfiguration of the networks in *tt4*. Metabolites with the asterisk (*) were quantified by using LC-Q-TOF/MS analysis (see Additional File 3). Abbreviations: CHS, chalcone synthase; *p*-Coumarate, 4-Hydroxycinnamic acid.

Among the metabolite correlation pairs significant in *tt4*, malate and sinapate exhibited a much tighter correlation (correlation coefficient: *r*_*Met *_= 0.89) than did WT (*r*_*Met *_= 0.50) and *mto1 *(*r*_*Met *_= 0.06) although these 2 metabolites are located at a distance in the metabolic map. Table 1 shows the correlation of malate or sinapate with the other metabolites belonging to the TCA cycle and shikimate pathways. Malate- citrate and malate- fumarate, which are components in the TCA cycle, exhibited significant correlations both in WT and *tt4*. On the other hand, malate and the aromatic compounds in the shikimate pathway (phenylalanine, tyramine, tyrosine, and sinapate) were correlated only in *tt4 *but not in WT (Table 1 and Figure 5) although the levels of these metabolites in *tt4 *were maintained at levels similar to those observed in WT (see Additional File 2). Among the compounds concerned with these unique correlations enforced in *tt4*, malate and sinapate are believed to be direct precursors for the formation of sinapoylmalate acting as a UV-B protectant [34,35]. Interestingly, the level of sinapoylmalate itself did not change in *tt4 *as compared with that in WT (Additional File 3). These results suggested that the correlations between malate- aromatic compounds were intensified in *tt4 *without any significant changes in the pools of metabolites concerned (Figure 5). The tight correlation between malate and sinapate may imply a connection with the role of sinapoylmalate against UV-B stress in the *tt4 *mutant, in which the UV-protective flavonoids are missing (see Discussion).

**Table 1:** Metabolite correlations focused on malate and sinapate.

Metabolite *X*	Metabolite *Y*	*r*_*Met *_in WT	*r*_*Met *_in *mto1*	*r*_*Met*_in *tt4*	Pathway
Malate	Maleate	0.80	0.46	0.91	TCA
	Succinate	0.70	-0.06	**0.89**	TCA
	Fumarate	0.83	0.07	0.90	TCA
	Citrate	0.92	0.79	0.87	TCA
	Phenylalanine	0.45	-0.01	**0.89**	Shikimate
	Shikimate	0.81	0.57	0.92	Shikimate
	Tyramine	0.44	0.10	**0.94**	Shikimate
	Tyrosine	0.48	0.48	**0.92**	Shikimate
	Sinapate	0.50	0.06	**0.89**	Shikimate
Sinapate	Maleate	0.74	0.59	**0.90**	TCA
	Succinate	0.85	0.74	0.86	TCA
	Fumarate	0.75	0.87	0.90	TCA
	Citrate	0.36	0.09	**0.84**	TCA
	Malate	0.50	0.06	**0.89**	Shikimate
	Phenylalanine	0.74	0.33	**0.86**	Shikimate
	Shikimate	0.72	0.38	**0.88**	Shikimate
	Tyramine	0.80	0.11	0.91	Shikimate
	Tyrosine	0.71	0.29	**0.89**	Shikimate

Boldface characters represent the unique correlations observed only in *tt4*. The unique correlations in *tt4 *are defined as *r*_*Met *_> 0.85 in *tt4 *and as *r*_*Met *_< 0.80 in WT and *mto1*. Abbreviations: TCA, tricarboxylic acid.

### Few metabolite correlations are conserved among WT, *mto1*, and *tt4 *mutants

**Figure 6:**
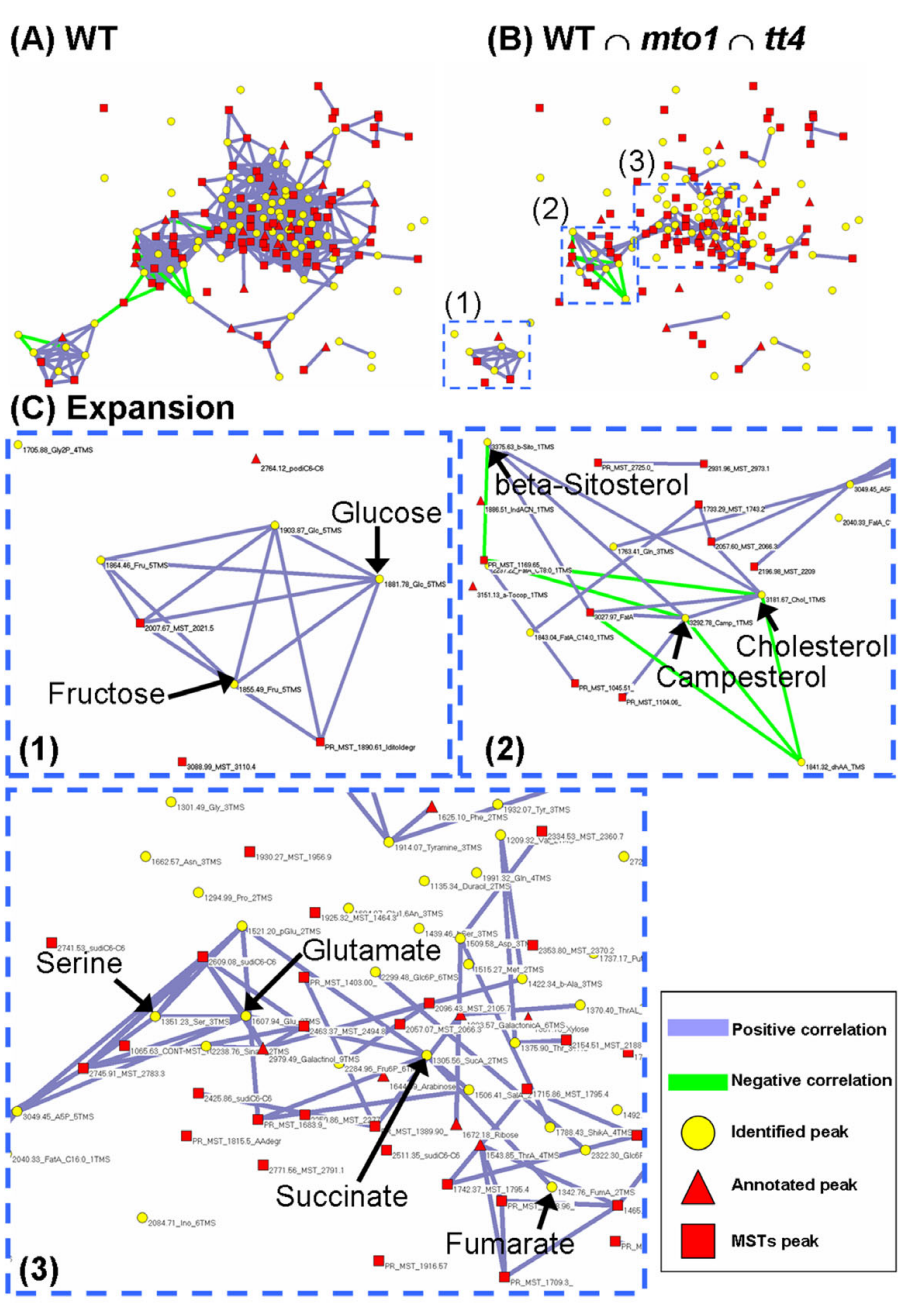
**The metabolite correlation networks**. (A) Significant metabolite correlation networks in WT plants (|*r*_*Met*_| ≥ 0.8, *n *= 17, *p *< 1.2 × 10^-4^). Each vertex corresponds to a metabolite or mass spectral tag (MSTs). The metabolites or MSTs with significant correlations are linked together. The colors of nodes represent the identified peaks (yellow circle), annotated peaks (red triangle) and MSTs (red square). The colors of the edges between nodes display the positive correlation (purple line) and the negative correlation (green line). (B) The common correlation pairs that are observed among WT, *mto1*, and *tt4*. (C) Expansions of the common network modules contain the following: (1) fructose-glucose, (2) cholesterol-campesterol and cholesterol-beta-sitosterol, and (3) succinate-fumarate and serine- glutamate.

Figure 6A presents the metabolite correlation network in WT. The metabolite correlation pairs conserved among WT, *mto1*, and *tt4 *are listed in Table 2 (only for identified metabolites) and are shown in Figure 6B (all the detected peaks). These correlation pairs conserved in the 3 genotypes are categorized into the following 3 types with respect to biosynthetic relations: (1) metabolite pairs placed close to each other in a metabolic pathway for the concerned reactions, e.g., the pairs of glucose-6-phosphate- fructose-6-phosphate, fructose- glucose, and the component pairs of the TCA cycle; (2) metabolite pairs possessing a similar chemical skeleton derived from the same biosynthetic pathway, e.g., the pairs of cholesterol, campesterol, and beta-sitosterol; (3) metabolite pairs located at a distance in a metabolic map, e.g., the pairs of valine- beta-alanine and serine- glutamate. Both valine and beta-alanine belong to the pantothenate (vitamin B_5_) synthesis process, although current knowledge regarding their regulatory relationship in this pathway in plants is limited. Serine and glutamate are involved in many metabolic reactions in various biosynthetic pathways and are directly connected by serine aminotransferase. These conserved metabolite correlations regardless of the mutations in *mto1 *and *tt4 *are presumed to reflect the fundamental metabolic regulations underlying plant metabolic systems. It is also notable that the network clusters in Figure 6 adequately represent the metabolic pathways, e.g., sucrose degradation in (1), steroids in (2), and the TCA cycle and amino acids in (3), indicating that the correlation network forms a modular structure, at least partly, according to the metabolic pathways.

**Table 2:** Significant correlations of identified metabolites conserved among 3 genotypes.

Metabolite *X*	Metabolite *Y*	*r*_*Met *_in WT	*p*	*r*_*Met *_in *mto1*	*p*	*r*_*Met *_in *tt4*	*p*
Fructose	Glucose	0.94	0.0000	0.94	0.0000	0.94	0.0000
Cholesterol	Campesterol	0.96	0.0000	0.97	0.0000	0.96	0.0000
Valine	beta-Alanine	0.92	0.0000	0.87	0.0001	0.96	0.0000
Lysine	Tyramine	0.91	0.0000	0.94	0.0000	0.98	0.0000
Serine	Glutamate	0.90	0.0000	0.87	0.0001	0.93	0.0000
Aspartate	Shikimate	0.90	0.0000	0.93	0.0000	0.97	0.0000
Campesterol	beta-Sitosterol	0.90	0.0000	0.98	0.0000	0.97	0.0000
Succinate	Glutamate	0.89	0.0000	0.85	0.0002	0.92	0.0000
Phenylalanine	Tyramine	0.88	0.0000	0.89	0.0001	0.95	0.0000
Succinate	Fumarate	0.87	0.0000	0.86	0.0002	0.86	0.0000
Glycerol-3-P	Inositol-1-P	0.85	0.0000	0.93	0.0000	0.75	0.0001
Isoleucine	Tyramine	0.84	0.0000	0.86	0.0002	0.90	0.0000
Glutamate	Adenosine-5-P	0.84	0.0000	0.86	0.0002	0.77	0.0001
Valine	Threonine	0.83	0.0000	0.88	0.0001	0.96	0.0000
Cholesterol	beta-Sitosterol	0.81	0.0001	0.98	0.0000	0.97	0.0000
Glutamine	Adenosine-5-P	0.81	0.0001	0.92	0.0000	0.83	0.0000

The significant level of the metabolite correlation was set at *p *< 0.001. Only pairs comprising identified metabolites are listed. The metabolites were defined as those identified on comparison with mass spectra and retention indices (RIs) from the library databases (Golm Metabolome Database [30] and our own library) and with authentic standards. Abbreviations: Glycerol-3-P, glycerol-3-phosphate; Inositol-1-P, *myo*-inositol-1-phosphate; Adenosine-5-P, adenosine-5-monophosphate.

### A new score for metabolites can select metabolomic markers of a mutant

In order to represent the metabolic characters of plants with different genotypes particularly in terms of metabolite correlation besides simple comparison of changes in metabolite levels, a new index for a metabolite representing the change of metabolite correlation was necessary. We therefore defined a new scoring method for metabolites that represents a diverse correlation in a genotype. This new criterion "z- score of metabolite correlation (ZMC)" represents the extent of uniqueness over given genotypes in terms of metabolite correlation, i.e., if metabolite correlation coefficient of metabolite *X *in a given genotype decreases markedly as compared to those in other genotypes, the ZMC of *X *in this genotype increases.

As shown in Table 3, ZMCs adequately delineated the key metabolites of each genotype. In *mto1*, methionine and methionine sulfone exhibited positive ZMCs as expected from their accumulation data. Spermidine showed a positive ZMC, suggesting that polyamine metabolism was affected in *mto1*. Interestingly, linoleate, linolenate, and ethanolamine showed negative ZMCs, indicating that the overall correlations of these metabolites were enhanced in *mto1*. Since these 3 metabolites are involved in glycerolipid biosynthesis, the network reconfiguration of the lipid metabolism may take place following methionine overaccumulation in *mto1*. In *tt4*, the sugar disaccharide exhibited a strongly positive ZMC, suggesting perturbation of sugar metabolism in *tt4 *as speculated from the slight changes in sugar-related metabolite levels. A number of metabolites extracted by ZMC were those annotated only by MSTs without identification. These metabolites are interesting for further characterization in order to understand the mechanism of network regulation in depth.

**Table 3:** Z-score of metabolite correlations (ZMC) found in (A) *mto1 *and (B) *tt4 *(*r*_*Met *_≥ 0.8).

**(A) Z-score in *mto1***			
**RI**	**Metabolite**	**Z-score**	**Significance**

1066	MST	3.45	≤0.001
1727	MST	2.56	≤0.05
1823	Methionine sulfone	2.42	≤0.05
2057	MST	2.07	≤0.05
2168	MST	2.07	≤0.05
1515	Methionine	1.85	≤0.10
2252	Spermidine	1.85	≤0.10
1645	Arabinose	1.71	≤0.10
2208	Linoleate	-1.72	≤0.10
2216	Linolenate	-1.72	≤0.10
1263	Ethanolamine	-1.72	≤0.10
1441	PR-MST	-1.72	≤0.10

**(B) Z-score in *tt4***			

**RI**	**Metabolite**	**Z-score**	**Significance**

2752	Sugar disaccharide	6.97	≤0.001
1104	PR-MST	1.99	≤0.05
2404	Inositol-1-phosphate	1.99	≤0.05
1773	PR-MST	1.81	≤0.10

Abbreviations: RI, retention index; MST, mass spectral tag obtained from the Golm Metabolome Database (GMD) [30]; PR-MST, Platform for RIKEN Metabolome (PRIMe)-mass spectral tag defined by our custom library for GC-MS spectra at PRIMe.

It should also be noted that the simple comparison of these metabolite levels listed by ZMC did not show significant differences except in the case of methionine and methionine sulfone. Thus, combinatory analyses of metabolite levels and metabolite correlation, such as ZMC, are important to obtain a clear insight into the manner in which the system is regulated and to generate a new hypothesis for characterizing metabolic systems in plants with different genotypes.

### Projection of metabolite correlation to gene-expression correlation gives clues on how metabolic networks are regulated

Since the conserved metabolite correlations across the different genotypes are presumed to characterize fundamental networks regardless of mutations, the investigation of gene expression in the metabolism of these metabolite pairs may lead to deeper insight into network regulation. The nature of metabolic networks represented as metabolite correlation might be explained, at least partly, by the correlation of gene expressions, and new findings regarding the gene-expression network could be obtained from the correlations of conserved metabolite.

**Figure 7:**
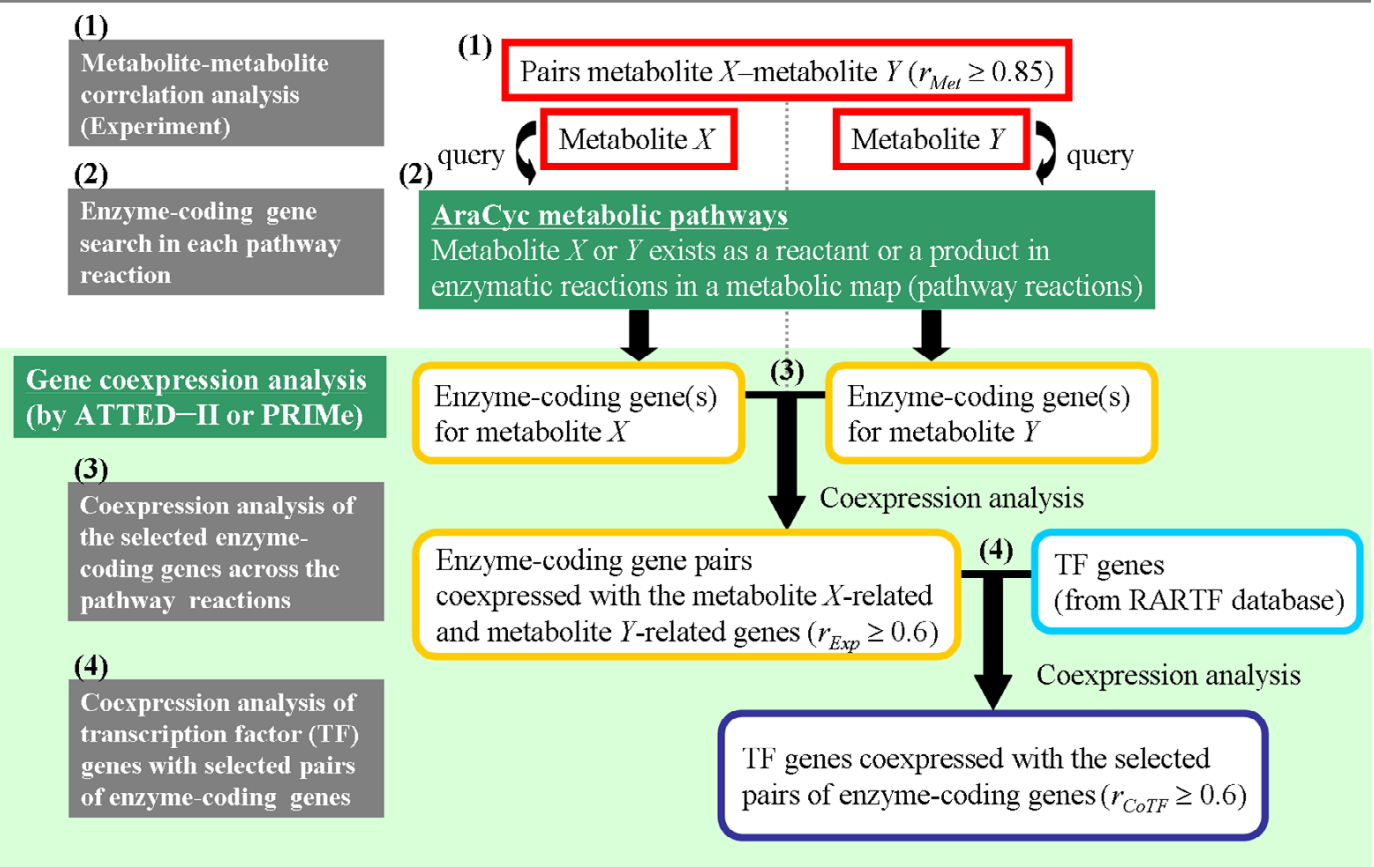
**Schematic flowchart of the coexpression analysis of genes based on metabolite correlations by using public transcriptome databases**. The process consists of 4 steps: (1) metabolite-metabolite correlation analysis, (2) extraction of enzyme-coding genes from the AraCyc pathways, (3) coexpression analysis of the extracted enzyme-coding gene pairs, and (4) coexpression analysis of genes encoding transcription factors with the coexpressed pairs of enzyme-coding genes. Abbreviations: *r*_*Exp*_, weighted Pearson's correlation coefficient of a pair of enzyme-coding genes for metabolites *X *and *Y*; *r*_*CoTF*_, weighted Pearson's correlation coefficient of the common transcription factor (TF) gene that is coexpressed with both the enzyme-coding genes of metabolites *X *and *Y*.

Figure 7 illustrates the flow chart of the mining of gene-expression correlation based on metabolite correlation pairs. Given metabolites *X *and *Y *as a correlating pair, we initially searched for genes coding for enzymes directly involved in the metabolic reactions of metabolite *X *or *Y *acting as a reactant or as a product in AraCyc metabolic map [36]. Subsequently, coexpression analysis among extracted enzyme-coding genes was performed by using the ATTED-II coexpression database of Arabidopsis transcriptome [22]. Coexpressed patterns (*r*_*Exp *_≥ 0.6) were observed in 49 gene-expression pairs across the whole transcriptome database (listed in Additional File 4). In order to exclude the pairs whose apparent coexpression pairs are simply due to their narrow tissue-specific expression patterns, we searched for Gene Atlas in Genevestigator [21] for the expression of condition specificity. Of these 49 enzyme-coding gene pairs, 6 coexpression pairs were indeed simply explained by tissue-specific expression patterns (for example, pollen and stamen), while the remaining 43 pairs of coexpression were most likely ascribed to their intrinsic coexpression natures, presumably causing the metabolite correlations. These gene pairs would prove interesting for further investigation to elucidate the bases of metabolic networks such as finding the transcription factors coexpressing with these enzyme-coding genes (as also shown in Figure 7). However, if we calculate the proportion of coexpressed enzyme-coding gene pairs over all the possible combinations of genes involved, only a very small fraction of combinations (0.2–3.9%) showed coexpression (Additional File 5) indicating the presence of multiple layers of regulation of the metabolic network not restricted to the level of gene expression. For further results see Additional file 6.

## Discussion

### Single deregulation of methionine synthesis disturbs the entire primary metabolism

For *mto1*, a significant increase in the level of homocysteine, methionine, and *S*-adenosyl-methionine was observed as expected from the overaccumulation of methionine; unexpectedly, there was a significant decrease in the levels of pipecolate and uracil (Figure 2A). Pipecolate is known to be a component of the lysine degradation pathway in a variety of organisms including mammals, fungi, and plants [37-39]. Uracil is derived from the aspartic acid family connected to methionine. In addition, there was a significant increase in the levels of arginine and ornithine. Arginine and ornithine belong to the glutamate family and are precursors of putrescine, which is an important metabolite in polyamine biosynthesis [40]. The results of ZMC analysis also indicated spermidine as an index metabolite of *mto1*. These data suggest that the overaccumulation of methionine affects not only methionine metabolism but also the regulation of the glutamate family leading to the regulation of polyamines. With regard to the correlations of metabolites belonging to the aspartate, glutamate, and polyamine biosynthetic pathways, most of the metabolites were highly conserved in both WT and *tt4 *but not in *mto1*. These results indicate that the perturbation in methionine metabolism causes a strong impact and a reconfiguration in the cellular nitrogen status by finely adjusting the metabolic networks of the nitrogenous metabolites.

### Backup system is reconfigured for protection against UV stress in the *tt4 *mutant

The metabolite pairs of the malate- shikimate pathways, in particular, the malate- sinapate pair, exhibited enhanced correlations in *tt4 *although the levels of these compounds remain unchanged (Figure 5). Sinapoylmalate, a major metabolite of sinapoylesters, is believed to play a protective role against UV-B in Arabidopsis [41]. The correlation observed between malate and sinapate in *tt4*, which is not observed in WT, implies the reconfiguration of metabolic networks lacking flavonoids in *tt4 *to efficiently produce an alternative defense compound, sinapoylmalate, thereby replacing the UV-protectant flavonoids. It is worthwhile to note that this correlation was observed even under normal conditions of light, in which no elevated accumulations of sinapoylmalate and its precursors were observed; this suggests the presence of a yet unidentified mechanism of cellular preparation for an adaptive response to UV stress by the reconfiguration of the metabolite networks in Arabidopsis. When we performed the UV-B stress experiment on WT and *tt4*, the levels of sinapoylmalate and sinapoylglucose were increased more in *tt4 *than in WT under UV irradiation (unpublished results). More detailed statistical and correlation analyses for sinapoylmalate, sinapoylglucose, and other aromatic compounds are in progress.

### A novel strategy for the prediction of genes responsible for metabolic networks by combined correlation analysis of metabolite accumulation and gene expression

There are a few reports available in literature concerning the correlations of metabolite accumulation, enzyme activities, and gene expression. The metabolite correlation, mainly in primary metabolites, is presumed to represent the direct and indirect interconnections in the metabolic pathway, even in the absence of causality between the correlation profiles and the pathway [18]. Non-synchronized patterns of transcripts and enzymes have been observed in WT and the starch-free mutant of Arabidopsis [42,43]. The significant metabolite-to-gene correlation is likely to reflect the effects of metabolic regulation that occur from metabolites to gene expression, rather than those from gene expression to metabolites [13]. The origin of high metabolite correlation is suggested to be derived either from the strong control of a single common enzyme between metabolites or from the increase in the level of a single enzyme as compared to the others [44].

Several studies indicate the power of integrated analysis of the metabolome and transcriptome [9-11], in particular, the feasibility of coexpression analysis by public transcriptome datasets [45,46], for gene characterization. Thus, we conceived that the basis of metabolic networks conserved across plants of different genotypes can be explained, at least partly, by the correlation of gene expression, and a better understanding of gene function can be obtained in the context of metabolic networks.

When we searched for the coexpression of enzyme-coding genes directly connected to metabolite pairs whose correlation is conserved, only a small fraction of genes were coexpressed. Nevertheless, we could filter the coexpressed enzyme-coding gene pairs (Additional File 4) that are presumably important for the regulation of metabolic networks. With regard to the serine-glutamate pair, *serine hydroxymethyltransferase 1 *(*SHM1*) (At4g37930) showed a strong correlation with *glutamate:glyoxylate aminotransferase 1 *(*GGT1*) (At1g23310). Both enzymes are involved in the photorespiration/metabolic salvage pathway, suggesting a coordinate regulation of this pathway at both the gene expression and metabolite levels. In addition, a number of genes of unknown function are also listed. It is intriguing to deduce the function of these genes by accounting for their correlation with metabolite pairs by further study.

Furthermore, we extended the coexpression analysis to the transcription-factor genes with the enzyme-coding genes (Figure 7). This search extracted *CONSTANTS-LIKE3 *(*COL3*) (At2g24790) and 3 unknown transcription factor genes (At1g68520, At4g36540, and At4g14540) from the metabolite pair of serine-glutamate. COL3 was previously identified as a constitutive photomorphogenetic 1 (COP1)-interacting protein and was suggested to be a positive regulator of light signaling [47]. Three unknown genes exhibited many connections with several enzyme-coding genes (*SHM1*, *GGT1*, *glutamine synthetase 2 *[*GS2*], and *glutamate-1-semialdehyde 2,1-aminomutase 2 *[*GSA2*]) involved in photorespiration/metabolic salvage pathway and nitrate assimilation. These unknown transcription-factor genes are of interest for further characterization because these may play an important role in the metabolic network of serine-glutamate. In addition to the serine-glutamate pairs, the other metabolite correlation pairs led to the suggestion of several transcription-factor genes through enzyme-coding gene coexpression in the same manner. This strategy can be a novel way of delimiting genes involved in metabolic networks.

## Conclusion

Metabolite profiling and subsequent metabolomic network analysis have been carried out for *A. thaliana *wild-type and 2 metabolic mutants – *mto1 *and *tt4 *– with the following conclusions.

1. We have established a widely applicable metabolome analysis pipeline adopting GC-TOF/MS with an H-MCR deconvolution method followed by metabolite annotation/identification, comparison of metabolite levels, and metabolite correlation analysis. For the metabolite correlation analysis, we proposed a new scoring method called ZMC to extract "index metabolites" representing the metabolome of a given genotype.

2. The overaccumulation of methionine affected not only the methionine-related and polyamine metabolisms but also the overall metabolic regulation in terms of metabolite levels and correlation networks.

3. Flavonoid-free mutation in *tt4 *had a small impact on the overall metabolic regulation. However, the malate-sinapate correlation strongly intensified, indicating a mechanism of metabolic reconfiguration to prepare a UV response in the absence of flavonoids.

4. By coexpression analysis based on metabolite correlations conserved across the 3 genotypes, genes that are likely involved in the cellular regulation of metabolic networks have been delimited.

## Methods

### Reagents

All chemicals excluding isotope reference compounds and reagents for silylation were purchased from Sigma Aldrich (Tokyo, Japan). The 6 stable isotope compounds ([^13^C_5_]-proline, [^2^H_4_]-succinic acid, [^2^H_6_]-2-hydroxybenzoic acid, [^13^C_3_]-myristic acid, [^13^C_12_]-sucrose, [^2^H_7_]-cholesterol) were purchased from Cambridge Isotope Laboratories (Andover, MA, USA), and [^13^C_5_, ^15^N] glutamic acid and [^13^C_6_]-glucose were obtained from Spectra Stable Isotopes (Columbia, Maryland, USA). [^2^H_4_]-1,4-diaminobutane was from C/D/N ISOTOPES (Pointe-Claire, Quebec, Canada). [^13^C_4_]-hexadecanoic acid was from Icon (Mt. Marion, NY, USA). The reagent for trimethylsilylation, *N*-methyl-*N*-trimethylsilyltrifluoroacetamide (MSTFA) with 1% of trimethylchlorosilane (TMCS), was purchased from Pierce (Rockford, IL, USA).

### Plant material and harvest

Wild-type *A*.*thaliana *plants accession Columbia (Col-0) and the mutants *mto1 *[23] and *tt4 *[48] of Col-0 background were obtained from Dr. Naito, Hokkaido University, and Dr. Kitamura, Japan Atomic Energy Research Institute, respectively. The sterilized seeds were stratified at 5°C for 2 d, and were successively sown on Murashige and Skoog (MS) medium containing 1% sucrose. Plants were cultivated in controlled growth chambers at 22°C in 16-h light and 8-h dark conditions for 18 d. The aerial regions were harvested with 20 different biological replicates, 6 h after the onset of the bright phase. Among these harvested plants, the plants that had fresh weight over 13 mg were used for GC-TOF/MS analysis (WT, *n *= 17; *mto1*, *n *= 13; and *tt4*, *n *= 20). For liquid chromatography-quadrupole-time-of-flight/mass spectrometry (LC-Q-TOF/MS) analysis, 3 biological replicates were prepared. All the plant materials were frozen immediately in liquid nitrogen to quench the enzymatic activity.

### Extraction and sample preparation for GC-TOF/MS analysis

Each sample was extracted with a concentration of 25 mg fresh weight of tissues per μl extraction medium (methanol/chloroform/water [3:1:1 v/v/v]) containing 10 stable isotope reference compounds using a Retsch mixer mill MM310 at a frequency of 30 Hz for 3 min at 4°C. Each isotope was adjusted to a final concentration of 22.5 ng per μl injection [49,50]. After centrifugation for 5 min at 15,100 × *g*, 400 μl of the supernatant was used for further analysis. The extracts were evaporated to dryness in a Savant SPD2010 SpeedVac Concentrator (Thermo Electron Corporation, Waltham, MA, USA).

For methoximation, 20 μl of methoxyamine hydrochloride (20 mg/ml in pyridine) was added to the sample. After 30 h of derivatization at room temperature, the sample was trimethylsilylated for 1 h using 20 μl of MSTFA with 1% TMCS at 37°C with shaking. Twenty μl of *n*-heptane was added following silylation. All the derivatization steps were performed in the vacuum glove box VSC-100 (Sanplatec, Japan) filled with 99.9995% (G3 grade) of dry nitrogen. The analysis of metabolites by GC-TOF/MS was performed as described previously [28].

### LC-Q-TOF/MS analysis

Frozen leaves were homogenized in 5-μl extraction solvent (methanol/H_2_O [4:1 v/v]) per mg fresh weight of tissues by using a mixer mill at a frequency of 20 Hz for 3 min at 4°C. After centrifugation at 12,000 × *g*, the cell debris was discarded, and the extracts were centrifuged again. These supernatants were immediately used for flavonoid analysis. For flavonoid profiling, Waters Acquity UPLC™ system (Waters Co., Massachusetts, USA) fitted with a Q-ToF Premier mass spectrometer (Micromass MS Technologies, Manchester, UK) was used. Ultra-performance liquid chromatography (UPLC) was carried out on a UPLC™ BEH C_18 _column (100-mm length × 2.1-mm inner diameter, 1.7-μm particles, Waters Co.) at a flow rate of 0.5 ml/min at 35°C. The elution gradient comprised solvent A (0.1% trifluoroacetic acid in H_2_O) and solvent B (0.1% trifluoroacetic acid in acetonitrile) and the elution profile – 0 min, 100% A; 5 min, 11% B; 20 min, 13% B; 24 min, 100% B – using linear gradients in between the time points. A photodiode array (PDA) detector was used for the detection of UV-visible absorption in the range of 210–500 nm. The TOF mass analyzer was used for the detection of flavonoid glycosides [M+H]^+ ^and fragment ions peak in a positive ion mode scan. The desolvation temperature was 450°C with a nitrogen gas flow rate of 600 l/h, capillary spray at 3.2 kV, source temperature at 150°C, and cone voltage at 35 V.

The peaks in the plant extracts were identified based on retention times, UV visible absorption spectra, and mass fragmentation by tandem MS analysis as reported [45]. The amounts of each kaempferol glycoside and sinapoyl derivative were calculated using kaempferol (at 340 nm) or sinapic acid (at 340 nm) as a standard, respectively.

### Processing of GC-TOF/MS data

Nonprocessed MS data from GC-TOF/MS analysis were exported in NetCDF format to MATLAB 6.5 (Mathworks, Natick, MA, USA), where all data-pretreatment procedures, such as smoothing, alignment, time-window setting, and H-MCR, were carried out [27]. The resolved MS spectra were matched against reference mass spectra using the NIST mass spectral search program for the NIST/EPA/NIH mass spectral library (version 2.0) and our custom software for peak annotation written in JAVA. Peaks were identified or annotated based on retention indices (RIs) and the reference mass spectra comparison to the Golm Metabolome Database (GMD) [30] and our in-house spectral library. The metabolites were identified by comparison with RIs from the library databases (GMD and our own library) and with those of authentic standards, and the metabolites were defined as annotated metabolites on comparison with mass spectra and RIs from these two libraries. The amount of *S*-adenosyl-methionine was calculated by the sum of the mass numbers at m/z 188 and 236 using Leco ChromaTOF software version 2.32 (LECO, St. Joseph, MI, USA) since this compound was not adequately detected by H-MCR.

### Statistical data analysis for metabolic profiling and metabolite correlation analysis

After peak annotation, the obtained data matrix (observations: biological replicates, variables: 171 annotated peaks) was used for statistical analyses including multivariate analysis and correlation analysis as log_10_-transformed data. Multivariate analysis was performed with SIMCA-P 11.0 software (Umetrics AB, Umeå, Sweden). Two types of multivariate analyses were applied: principal component analysis (PCA) as an unsupervised analysis and partial least square-discriminate analysis (PLS-DA) as a supervised analysis (WT, *n *= 17; *mto1*, *n *= 16 [13 samples from biological replicates and 3 samples from analytical replicates]; and *tt4*, *n *= 20). The PCA was applied to check the sample distribution before performing PLS-DA. In order to narrow down the loadings that make a contribution to separate the groups in PLS-DA, the loading of principal component 1 in each model was chosen according to the value of the first weight vector (w* 1) together with the 99% confidence intervals calculated using jack-knifing [51,52]. Statistical tests were performed using R package, which is an open source statistical computing environment [54]. Simple comparisons of the means of the obtained peak areas were performed by Welch's *t-*test. A difference with *p *< 0.05 was considered to be significant. Pair-wise metabolite- metabolite correlations were calculated by Pearson's correlation coefficient (*r*_*Met*_) test using 171 peaks. The level of significance was determined as follows: |*r*_*Met*_| ≥ 0.8 (*p *< 1.2 × 10^-4^, *n *= 17) for WT, |*r*_*Met*_| ≥ 0.85 (*p *< 2.3 × 10^-4^, *n *= 13) for *mto1*, and |*r*_*Met*_| ≥ 0.75 (*p *< 1.4 × 10^-4^, *n *= 20) for *tt4*. The network of pair-wise metabolite correlation is drawn using Pajek [55].

ZMC was calculated as follows: (1) For a metabolite in a given genotype (e.g., *mto1*), all the significant metabolite correlation pairs (e.g. *r*_*Met *_≥ 0.8) which contained the metabolite as a pair in 2 genotypes (e.g., WT and *tt4*) were chosen; (2) For the pairs selected in step (1), the average correlation coefficient in the 2 genotypes (e.g., WT and *tt4*) was calculated; (3) The difference in the correlation coefficient between a given genotype and the other 2 genotypes was obtained by subtracting the average coefficient in 2 genotypes from the coefficient of a given genotype; (4) The z-score of each relative correlation obtained was calculated and defined as a ZMC. Finally, the ZMCs across all pairs were computed. Given that ZMC follows normal distribution, the ZMC of a metabolite is regarded as an index representing the characteristic metabolotype in terms of metabolite correlation in a given genotype.

### Coexpression analysis of genes using publicly available data source

In order to understand the relationship between metabolite-metabolite correlations and the coexpression patterns of the genes encoding collateral enzymes for each concerned metabolite, publicly available data that were obtained from AraCyc database version 3.5 [36] as a text format dump file "AraCyc Pathways" that listed accessions for 262 different pathways associated with 1885 genes (aracyc_dump_20070213) and another file "AraCyc Compounds" that contained all compounds found in the AraCyc pathways (aracyc_compounds_20070213) were used. When metabolite *X *(or metabolite *Y*) reacts with an enzyme as a substrate or a product, the enzyme was defined as a "reaction" enzyme. The coexpression analysis was performed between the enzyme-coding genes for metabolite *X *and metabolite *Y*. In order to search for coexpressed genes, we used a tool named "Correlated Gene Search" from the website [56] released by our institute (to be published elsewhere). The expression data were produced using Affymetrix GeneChip by AtGenExpress at the Arabidopsis information resource (TAIR), and the correlation data has been released in the ATTED-II database [22]. The search conditions were as follows: the type of matrix was "all dataset ver. 3" since the source of GeneChip data were the 58 experiments including 1,388 arrays; threshold = 0.6, method = "interconnection of sets", display limit = 99999 (maximum). The distribution of the correlation coefficient for coexpressed genes via ATTED-II followed the normal distribution (the standard deviation was equal to 0.18). The result suggested that a threshold above 0.6 could correspond with 3 standard deviations.

For a coexpression analysis of the transcription factor and coexpressed enzyme-coding gene pairs across the reactions for each metabolite, a list of 1968 transcription-factor genes was made from RIKEN Arabidopsis Transcription Factor database (RARTF) [53]. Using AGI code numbers in the list, coexpression analysis was performed using the tool "Correlated Gene Search" from the website [56]. The organ-specificity of the expression of the coexpressed genes was investigated *in silico *by using Genevestigator [21].

## Authors' contributions

MK and AF contributed equally to this work; MK, AF, TT, and KS designed the research; MK, AF, MKobayashi, NH, and TT performed research; MK, AF, MA, PJ, and TM analyzed data; and MK, AF, MA, and KS wrote the paper. All authors read and approved the final manuscript.

## Supplementary Material

Additional file 1 List of metabolites that contributed to the separation of the mutant profiles group from those of wild-type (WT). Discriminative metabolites of the first component in the PLS-DAs for *mto1 *or *tt4 *against WT are shown. Click here for file

Additional file 2 List of metabolic changes represented by x-fold (mutant/WT) and *p*-value. A comprehensive list of all measured peaks and the changes of these levels are presented. Boldface characters represent the significant differences (*p *< 0.05). Click here for file

Additional file 3 Changes in metabolite concentration of flavonols and sinapoylesters in WT and *tt4 *plants. Click here for file

Additional file 4 Pairs of coexpressed enzyme-coding genes for direct production/degradation of metabolites *X *and for *Y*. Click here for file

Additional file 5 Proportionality of coexpressed enzyme-coding gene pairs related to metabolic reactions of metabolite pairs conserved in three genotypes. Click here for file

Additional file 6 Data matrix used in the present study. Normalized data matrix of peak areas of extracted MS spectra for 50 individual plants (17 × WT, 13 × *mto1*, and 20 × *tt4*). Click here for file
