# Supplementary material for: Unbiased characterization of genotype-dependent metabolic regulations by metabolomic approach in Arabidopsis thaliana
Source: BMC Syst Biol. 2007 Nov 21;1:53. doi: 10.1186/1752-0509-1-53 (PMC2233643; doi:10.1186/1752-0509-1-53)
Supplement: Additional file 1 — List of metabolites that contributed to the separation of the mutant profiles group from those of wild-type (WT). Discriminative metabolites of the first component in the PLS-DAs for mto1 or tt4 against WT are shown. [file 1752-0509-1-53-S1.doc]

## Discriminative metabolites of the first component in the PLS-DAs for *mto1* or *tt4* against WT.

**A) *mto1*/WT**

| RI | Metabolite | PC1 loadings (*mto1*/WT separation) | x-fold (*mto1*/WT) | *p* |
| --- | --- | --- | --- | --- |
| 1332 | Uracil | 0.141 | 0.47 | 0.0000 |
| 1439 | Homoserine | -0.133 | 1.34 | 0.0000 |
| 1476 | Malate | -0.140 | 1.59 | 0.0000 |
| 1515 | Methionine | -0.181 | 34.12 | 0.0000 |
| 1656 | Homocysteine | -0.181 | 43.59 | 0.0000 |
| 1808 | Ornithine | -0.150 | 2.27 | 0.0000 |
| 1813 | Arginine^a^ | -0.152 | 2.33 | 0.0000 |
| 1823 | Methionine sulfone | -0.174 | 12.24 | 0.0000 |
| 1990 | *N*-acetyl-Ornithine | -0.124 | 1.91 | 0.0000 |
| 2285 | Fructose-6-P | -0.158 | 2.06 | 0.0000 |
| 2299 | Glucose-6-P | -0.128 | 1.45 | 0.0000 |
| 3151 | alpha-Tocopherol | -0.117 | 1.75 | 0.0000 |
| 2426 | sudiC6-C6^*^ | -0.120 | 1.45 | 0.0001 |
| 1788 | Shikimate | -0.117 | 1.23 | 0.0002 |
| 1343 | Fumarate | -0.105 | 1.33 | 0.0005 |
| 1376 | Threonine | 0.103 | 0.79 | 0.0009 |
| 1135 | Dihydrouracil | 0.099 | 0.77 | 0.0010 |
| 1493 | Erythritol | -0.105 | 1.22 | 0.0012 |
| 1695 | 1,6-anhydro- Glucose | -0.109 | 1.21 | 0.0014 |
| 1264 | Phosphate | -0.102 | 1.29 | 0.0016 |
| 1645 | Arabinose | -0.105 | 1.19 | 0.0018 |
| 1672 | Ribose | -0.096 | 1.17 | 0.0035 |
| 1364 | Pipecolate | 0.09 | 0.40 | 0.0041 |
| 1422 | beta-Alanine | -0.096 | 1.20 | 0.0041 |
| 2728 | Trehalose | -0.091 | 1.25 | 0.0048 |
| 2903 | sudiC6-C6* | -0.097 | 1.22 | 0.0083 |
| 1484 | Threitol | 0.096 | 0.88 | 0.0119 |
| 2050 | Sinapate | 0.081 | 0.88 | 0.0125 |
| 2040 | FatA C16:0 | 0.089 | 0.96 | 0.0145 |
| 1932 | Tyrosine | 0.069 | 0.79 | 0.0173 |
| 1287 | Isoleucine | -0.078 | 1.13 | 0.0311 |
| 1263 | Ethanolamine | -0.075 | 1.16 | 0.0344 |
| 1319 | Glycerate | -0.077 | 1.16 | 0.0410 |
| 1625 | Phenylalanine | -0.071 | 1.13 | 0.0412 |

^*^ MST annotated peaks.

^a^ Arginine was detected as *N*-guanylproline.

**(B) *tt4***/WT

| RI | Metabolite | PC1 loadings (*tt4*/WT separation) | x-fold (*tt4*/WT) | *p* |
| --- | --- | --- | --- | --- |
| 1370 | *trans*-Threonic acid-1,4-lactone | 0.134 | 0.73 | 0.0000 |
| 1544 | Threonate | 0.135 | 0.67 | 0.0000 |
| 1264 | Phosphate | -0.137 | 1.40 | 0.0001 |
| 1891 | PR-MST* | 0.132 | 0.51 | 0.0001 |
| 1948 | Galactitol | 0.131 | 0.80 | 0.0001 |
| 2239 | Sinapate | 0.128 | 0.70 | 0.0001 |
| 2619 | Sucrose | 0.133 | 0.69 | 0.0001 |
| 2764 | podiC6-C6* | 0.132 | 0.46 | 0.0001 |
| 2085 | *myo*-Inositol | 0.119 | 0.79 | 0.0004 |
| 2522 | sudiC6-C6* | 0.116 | 0.83 | 0.0005 |
| 2979 | Galactinol | 0.115 | 0.76 | 0.0006 |
| 1047 | Lactate | 0.118 | 0.64 | 0.0008 |
| 2568 | Nicotianamine | -0.117 | 1.33 | 0.0009 |
| 1788 | Shikimate | 0.109 | 0.79 | 0.0011 |
| 1984 | Galactonate | 0.110 | 0.77 | 0.0011 |
| 1343 | Fumarate | 0.107 | 0.73 | 0.0015 |
| 1706 | Glycerol-2-P | -0.110 | 1.59 | 0.0015 |
| 3365 | Raffinose | 0.106 | 0.68 | 0.0017 |
| 1476 | Malate | 0.098 | 0.76 | 0.0045 |
| 1882 | Glucose | 0.099 | 0.76 | 0.0047 |
| 1484 | Threitol | 0.094 | 0.85 | 0.0060 |
| 1319 | Glycerate | 0.093 | 0.76 | 0.0069 |
| 1645 | Arabinose | 0.092 | 0.80 | 0.0069 |
| 1296 | Maleate | 0.095 | 0.84 | 0.0071 |
| 1803 | Citrate | 0.094 | 0.75 | 0.0073 |
| 1855 | Fructose | 0.090 | 0.59 | 0.0096 |
| 1914 | Tyramine | 0.085 | 0.85 | 0.0142 |
| 1672 | Ribose | 0.077 | 0.85 | 0.0253 |
| 1493 | Erythritol | 0.073 | 0.87 | 0.0348 |
| 2728 | Trehalose | 0.075 | 0.82 | 0.0355 |
| 1439 | Homoserine | 0.073 | 0.88 | 0.0409 |

* MST annotated peaks.

Discriminative metabolites were extracted according to the value of the first weight vector (w* 1) in addition to the 99% confidence intervals calculated using jack-knifing. The extracted metabolites were further filtered using Welch’s *t*-test setting *p* < 0.05 as significance. The negative values of loading are of metabolites discriminative for mutants, whereas positive values are of metabolites discriminative for WT. The peaks are identified or annotated by using the PRIMe in-house mass spectral library and public mass spectral and retention index (MSRI) libraries at GMD [30]. Abbreviations: Fructose-6-P, fructose-6-phosphate; Glucose-6-P, glucose-6-phosphate; sudiC6-C6, sugar disaccharide C6-C6; FatA C16:0, *n*-hexadecanoic acid; PR-MST, Platform for RIKEN Metabolome (PRIMe)-mass spectral tag; podiC6-C6, polyol dimer C6-C6; Glycerol-2-P, glycerol-2-phosphate.
