# Supplementary material for: Unbiased characterization of genotype-dependent metabolic regulations by metabolomic approach in Arabidopsis thaliana
Source: BMC Syst Biol. 2007 Nov 21;1:53. doi: 10.1186/1752-0509-1-53 (PMC2233643; doi:10.1186/1752-0509-1-53)
Supplement: Additional file 3 — Changes in metabolite concentration of flavonols and sinapoylesters in WT and tt4 plants. [file 1752-0509-1-53-S3.doc]

## Changes in metabolite concentration of flavonols and sinapoylesters in WT and *tt4* plants.

|  | WT |  | *tt4* |  |
| --- | --- | --- | --- | --- |
| Metabolite | nmol/g F.W. | SD (*n* = 3) | nmol/g F.W. | SD (*n* = 3) |
| Kaempferol 3-Rha 7-Rha | 90.9 | ±7.3 | ND | ND |
| Kaempferol 3-Glc 7-Rha | 42.6 | ±0.5 | ND | ND |
| Kaempferol 3-Glc-2’’-Rha 7-Rha | 73.9 | ±7.3 | ND | ND |
| Sinapoylmalate | 1343.8 | ±25.3 | 1156.7 | ±55.7 |
| Sinapoylglucose | ND | ND | ND | ND |

Three flavonols and two sinapoylesters were measured by LC-Q-TOF/MS analysis. Abbreviations: F.W., flesh weight; SD, standard deviation; ND, not detected; Kaempferol 3-Rha 7-Rha, kaempferol 3-*O*-rhamnoside 7-*O*-rhamnoside; Kaempferol 3-Glc 7-Rha, kaempferol 3-*O*-glucoside 7-*O*-rhamnoside; Kaempferol 3-Glc-2’’-Rha 7-Rha, kaempferol 3-*O*-[rhamnosyl(1→2)-glucoside] 7-*O*-rhamnoside
