# Supplementary material for: Unbiased characterization of genotype-dependent metabolic regulations by metabolomic approach in Arabidopsis thaliana
Source: BMC Syst Biol. 2007 Nov 21;1:53. doi: 10.1186/1752-0509-1-53 (PMC2233643; doi:10.1186/1752-0509-1-53)
Supplement: Additional file 4 — Pairs of coexpressed enzyme-coding genes for direct production/degradation of metabolites X and for Y. [file 1752-0509-1-53-S4.doc]

## Pairs of coexpressed enzyme-coding genes for direct production/degradation of metabolites *X* and for *Y*.

| *r_Exp_* | Gene *X* | Gene name *X* | Tissue specificity of gene *X* | Description of gene *X* | Gene *Y* | Gene name *Y* | Tissue specificity of gene *Y* | Description of gene *Y* |
| --- | --- | --- | --- | --- | --- | --- | --- | --- |
| **Fructose−Glucose** | |  |  |  |  |  |  |  |
| 0.9 | At5g49190 | *SUS2* | Seed, silique | Encodes a sucrose synthase that is induced specifically by O(2) deficiency. | At5g09640 | *SNG2* | Seed, silique | encodes a serine carboxypeptidase-like (SCPL) protein |
| 0.71 | At4g26390 | AT4G26390.1 | Pollen, stamen | pyruvate kinase, putative | At3g05820 | AT3G05820.1 | Pollen, stamen | beta-fructofuranosidase, putative |
|  |  |  |  |  |  |  |  |  |
| **Serine−Glutamate** | | |  |  |  |  |  |  |
| 0.8 | At4g37930 | *SHM1* | Universal | Encodes a protein with mitochondrial serine hydroxymethyltransferase activity | At1g23310 | *GGT1* | Universal | glutamate:glyoxylate aminotransferase 1 (GGT1) |
| 0.8 | At4g37930 | *SHM1* | Universal | Encodes a protein with mitochondrial serine hydroxymethyltransferase activity | At5g35630 | *GS2* | Universal | chloroplastic glutamine synthetase |
| 0.8 | At5g27470 | AT5G27470.1 | Universal | seryl-tRNA synthetase / serine--tRNA ligase | At3g27740 | *CARA* | Universal | carbamoyl phosphate synthetase small subunit mRNA (carA) |
| 0.77 | At4g32520 | *SHM3* | Universal | serine hydroxymethyltransferase 3 | At1g63660 | AT1G63660.2 | Universal | GMP synthase (glutamine-hydrolyzing), putative |
| 0.75 | At5g27470 | AT5G27470.1 | Universal | seryl-tRNA synthetase / serine--tRNA ligase | At1g63660 | AT1G63660.2 | Universal | GMP synthase (glutamine-hydrolyzing), putative |
| 0.75 | At4g32520 | *SHM3* | Universal | serine hydroxymethyltransferase 3 | At3g27740 | *CARA* | Universal | carbamoyl phosphate synthetase small subunit mRNA (carA) |
| 0.72 | At1g56500 | AT1G56500.1 | Aerical parts | haloacid dehalogenase-like hydrolase family protein | At1g23310 | *GGT1* | Universal | glutamate:glyoxylate aminotransferase 1 (GGT1) |
| 0.71 | At1g11870 | *ATSRS* | Universal | Seryl-tRNA synthetase targeted to chloroplasts and mitochondria | At3g48730 | AT3G48730.1 | Universal | glutamate-1-semialdehyde 2,1-aminomutase 2 (GSA 2) |
| 0.71 | At1g56500 | AT1G56500.1 | Aerical parts | haloacid dehalogenase-like hydrolase family protein | At5g35630 | *GS2* | Universal | chloroplastic glutamine synthetase |
| 0.71 | At3g10050 | *OMR1* | Universal | first enzyme in the biosynthetic pathway of isoleucine | At3g27740 | *CARA* | Universal | carbamoyl phosphate synthetase small subunit mRNA (carA) |
| 0.71 | At3g10050 | *OMR1* | Universal | first enzyme in the biosynthetic pathway of isoleucine | At4g26900 | AT4G26900.1 | Universal | imidazole glycerol phosphate synthase hisHF, chloroplast |
| 0.7 | At1g56500 | AT1G56500.1 | Aerical parts | haloacid dehalogenase-like hydrolase family protein | At3g48730 | AT3G48730.1 | Universal | glutamate-1-semialdehyde 2,1-aminomutase 2 (GSA 2) |
| 0.7 | At4g32520 | *SHM3* | Universal | serine hydroxymethyltransferase 3 | At4g26900 | AT4G26900.1 | Universal | imidazole glycerol phosphate synthase hisHF, chloroplast |
| 0.69 | At4g32520 | *SHM3* | Universal | serine hydroxymethyltransferase 3 | At4g33680 | AT4G33680.1 | Universal | aminotransferase class I and II family protein |
| 0.69 | At3g10050 | *OMR1* | Universal | first enzyme in the biosynthetic pathway of isoleucine | At1g63660 | AT1G63660.2 | Universal | GMP synthase (glutamine-hydrolyzing), putative |
| 0.68 | At5g27470 | AT5G27470.1 | Universal | seryl-tRNA synthetase / serine--tRNA ligase | At4g26900 | AT4G26900.1 | Universal | imidazole glycerol phosphate synthase hisHF, chloroplast |
| 0.66 | At5g27470 | AT5G27470.1 | Universal | seryl-tRNA synthetase / serine--tRNA ligase | At4g33680 | AT4G33680.1 | Universal | aminotransferase class I and II family protein |
| 0.65 | At4g37930 | *SHM1* | Universal | Encodes a protein with mitochondrial serine hydroxymethyltransferase activity | At3g48730 | AT3G48730.1 | Universal | glutamate-1-semialdehyde 2,1-aminomutase 2 (GSA 2) |
| 0.63 | At4g13890 | *SHM5* | Universal | serine hydroxymethyltransferase 5 | At2g17630 | AT2G17630.1 | Universal | phosphoserine aminotransferase, putative |
| 0.63 | At4g32520 | *SHM3* | Universal | serine hydroxymethyltransferase 3 | At2g37500 | AT2G37500.1 | Universal | arginine biosynthesis protein ArgJ family |
| 0.63 | At1g56500 | AT1G56500.1 | Aerical parts | haloacid dehalogenase-like hydrolase family protein | At3g55630 | *ATDFD* | Aerial parts | *A. thaliana* DHFS-FPGS homolog D |
| 0.62 | At1g56500 | AT1G56500.1 | Aerical parts | haloacid dehalogenase-like hydrolase family protein | At2g13360 | AT2G13360.2 | Aerial parts | serine-glyoxylate aminotransferase-related |
| 0.62 | At3g10050 | *OMR1* | Universal | first enzyme in the biosynthetic pathway of isoleucine | At5g10240 | AT5G10240.2 | Universal | similar to asparagine synthetase 1 (glutamine-hydrolyzing) |
| 0.61 | At3g10050 | *OMR1* | Universal | first enzyme in the biosynthetic pathway of isoleucine | At4g33680 | AT4G33680.1 | Universal | aminotransferase class I and II family protein |
| 0.6 | At1g11870 | *ATSRS* | Universal | Seryl-tRNA synthetase targeted to chloroplasts and mitochondria | At3g10160 | *ATDFC* | Universal | *A. thaliana* DHFS-FPGS homolog C |
| 0.6 | At1g11870 | *ATSRS* | Universal | Seryl-tRNA synthetase targeted to chloroplasts and mitochondria | At1g63680 | *PDE316* | Universal | mutant has Pale seeds and seedlings |
| 0.6 | At3g22460 | AT3G22460.1 | Universal | cysteine synthase, putative | At5g46180 | *DELTA-OAT* | Universal | ornithine delta-aminotransferase |
|  |  |  |  |  |  |  |  |  |
| **Aspartate−Shikimate** | | |  |  |  |  |  |  |
| 0.73 | At3g13490 | AT3G13490.1 | Universal | tRNA synthetase class II (D, K and N) family protein | At2g35500 | AT2G35500.1 | Universal | shikimate kinase-related |
| 0.7 | At4g33760 | AT4G33760.1 | Universal | tRNA synthetase class II (D, K and N) family protein | At2g35500 | AT2G35500.1 | Universal | shikimate kinase-related |
|  |  |  |  |  |  |  |  |  |
| **Succinate−Glutamate** | | |  |  |  |  |  |  |
| 0.73 | At5g63590 | AT5G63590.1 | Roots | flavonol synthase, putative | At2g48140 | AT2G48140.1 | Roots, seed | protease inhibitor |
| 0.71 | At5g63600 | AT5G63600.2 | Roots | similar to flavonol synthase 1 (FLS1) | At5g17330 | *GAD* | Roots | Encodes one of two isoforms of glutamate decarboxylase. |
| 0.66 | At4g10490 | AT4G10490.1 | Silique, seed | oxidoreductase, 2OG-Fe(II) oxygenase family protein | At1g48470 | *GLN1;5* | Stamen, seed, flower | Encodes cytosolic glutamine synthase isozyme |
| 0.66 | At5g07200 | *YAP169* | Silique, seed | encodes a gibberellin 20-oxidase. | At1g48470 | *GLN1;5* | Stamen, seed, flower | Encodes cytosolic glutamine synthase isozyme |
| 0.62 | At1g79440 | AT1G79440.1 | Universal | succinate-semialdehyde dehydrogenase (SSADH1) | At5g60540 | AT5G60540.1 | Universal | Encodes a protein predicted to function in tandem with PDX1 |
| 0.6 | At2g24270 | AT2G24270.2 | Aerial parts | NADP-dependent glyceraldehyde-3-phosphate dehydrogenase, putative | At3g48730 | AT3G48730.1 | Universal | glutamate-1-semialdehyde 2,1-aminomutase 2 (GSA 2) |
| 0.6 | At3g50210 | AT3G50210.3 | Universal | similar to 2-oxoacid-dependent oxidase, putative (DIN11) | At5g37600 | *ATGSR1* | Universal | encodes a cytosolic glutamine synthetase |
|  |  |  |  |  |  |  |  |  |
| **Succinate−Fumarate** | | |  |  |  |  |  |  |
| 0.68 | At3g47833 | AT3G47833.1 | Universal | expressed protein | At1g08480 | AT1G08480.1 | Universal | expressed protein |
| 0.66 | At3g47833 | AT3G47833.1 | Universal | expressed protein | At2g46390 | AT2G46390.1 | Universal | expressed protein |
| 0.61 | At1g08480 | AT1G08480.1 | Universal | expressed protein | At2g46390 | AT2G46390.1 | Universal | expressed protein |
|  |  |  |  |  |  |  |  |  |
| **Valine−Threonine** | | |  |  |  |  |  |  |
| 0.82 | At5g49030 | AT5G49030.2 | Universal | similar to isoleucyl-tRNA synthetase, putative | At5g52520 | AT5G52520.1 | Universal | tRNA synthetase class II (G, H, P and S) family protein |
| 0.81 | At4g04350 | AT4G04350.1 | Universal | leucyl-tRNA synthetase, putative | At5g52520 | AT5G52520.1 | Universal | tRNA synthetase class II (G, H, P and S) family protein |
| 0.76 | At5g16715 | AT5G16715.1 | Universal | tRNA synthetase class I (I, L, M and V) family protein | At5g52520 | AT5G52520.1 | Universal | tRNA synthetase class II (G, H, P and S) family protein |
| 0.71 | At4g10320 | AT4G10320.1 | Universal | isoleucyl-tRNA synthetase, putative | At3g62120 | AT3G62120.2 | Universal | tRNA synthetase class II (G, H, P and S) family protein |
| 0.68 | At4g10320 | AT4G10320.1 | Universal | isoleucyl-tRNA synthetase, putative | At5g26830 | AT5G26830.1 | Universal | threonyl-tRNA synthetase / threonine--tRNA ligase (THRRS) |
| 0.65 | At1g14610 | *TWN2* | Universal | Required for proper proliferation of basal cells | At3g62120 | AT3G62120.2 | Universal | tRNA synthetase class II (G, H, P and S) family protein |
| 0.63 | At1g09620 | AT1G09620.1 | Universal | similar to leucyl-tRNA synthetase, putative | At3g62120 | AT3G62120.2 | Universal | tRNA synthetase class II (G, H, P and S) family protein |
| 0.63 | At1g09620 | AT1G09620.1 | Universal | similar to leucyl-tRNA synthetase, putative | At3g10050 | *OMR1* | Universal | first enzyme in the biosynthetic pathway of isoleucine |

The highly coexpressed genes (*r_Exp_* ≥ 0.6) are listed.

Abbreviation: *r_Exp_*, Weighted Pearson’s correlation coefficient of a pair of genes that contain predictive coding sequences for the production of enzymes for metabolites *X* and *Y*.
