# Supplementary material for: Unbiased characterization of genotype-dependent metabolic regulations by metabolomic approach in Arabidopsis thaliana
Source: BMC Syst Biol. 2007 Nov 21;1:53. doi: 10.1186/1752-0509-1-53 (PMC2233643; doi:10.1186/1752-0509-1-53)
Supplement: Additional file 5 — Proportionality of coexpressed enzyme-coding gene pairs related to metabolic reactions of metabolite pairs conserved in three genotypes. [file 1752-0509-1-53-S5.doc]

## Proportionality of coexpressed enzyme-coding gene pairs related to metabolic reactions of metabolite pairs conserved in 3　genotypes.

| Common metabolite pair | |  |  |  |  |  | Ratio (%) |
| --- | --- | --- | --- | --- | --- | --- | --- |
| Metabolite *X* | Metabolite *Y* | (A) | (B) | (C) | (D) | (E) | (A)/(E) |
| Fructose | Glucose | 2 | 21 | 6 | 12 | 450 | 0.4 |
| Glutamate | Galactinol | 1 | 91 | 7 | 0 | 637 | 0.2 |
| Serine | Glutamate | 27 | 35 | 91 | 1 | 3311 | 0.8 |
| Aspartate | Shikimate | 2 | 24 | 10 | 0 | 240 | 0.8 |
| Succinate | Glutamate | 7 | 48 | 91 | 0 | 4368 | 0.2 |
| Succinate | Fumarate | 3 | 37 | 6 | 11 | 695 | 0.4 |
| Valine | Threonine | 8 | 15 | 12 | 1 | 207 | 3.9 |

The number of coexpressed enzyme-coding gene pairs for the metabolite pair *X* and *Y* calculated using the public database ATTED-II is shown in (A). Enzyme-coding genes for metabolite *X* or *Y* were extracted based on the classification of enzymatic reactions in AraCyc map. The coexpressed gene pairs with *r_Exp_* ≥ 0.6 were filtered and counted. (B) the number of genes related to metabolite *X*; (C) the number of genes to metabolite *Y*; (D) the number of common genes for metabolite *X* and *Y*; (E) the sum of all the possible combinations between (B) and (C), (B) and (D), and (C) and (D).

Abbreviation: *r_Exp_*, weighted Pearson’s correlation coefficient provided from ATTED-II.
